# A Tubing-Free Microfluidic Wound Healing Assay Enabling the Quantification of Vascular Smooth Muscle Cell Migration

**DOI:** 10.1038/srep14049

**Published:** 2015-09-14

**Authors:** Yuanchen Wei, Feng Chen, Tao Zhang, Deyong Chen, Xin Jia, Junbo Wang, Wei Guo, Jian Chen

**Affiliations:** 1 State Key Laboratory of Transducer Technology, Institute of Electronics, Chinese Academy of Sciences, Beijing, P.R. China, 100190; 2 Department of Vascular Surgery, Clinical Division of Surgery, Chinese PLA General Hospital, Beijing, P.R. China, 100853; 3 Department of Vascular Surgery, Peking University People’s Hospital, Beijing, P.R. China, 100044

## Abstract

This paper presents a tubing-free microfluidic wound healing assay to quantify the migration of vascular smooth muscle cells (VSMCs), where gravity was used to generate a laminar flow within microfluidic channels, enabling cell seeding, culture, and wound generation. As the first systemic study to quantify the migration of VSMCs within microfluidic environments, the effects of channel geometries, surface modifications and chemokines on cellular migration were investigated, revealing that 1) height of the micro channels had a significant impact on cell migration; 2) the surface coating of collagen induced more migration of VSMCs than fibronectin coated surfaces and 3) platelet derived growth factor resulted in maximal cell migration compared to tumor necrosis factor alpha and fetal bovine serum. Furthermore, migrations of five types of VSMCs (e.g., the human vascular smooth muscle cell line, two types of primary vascular smooth cells, and VSMCs isolated from two human samples) were quantified, finding that VSMCs from the cell line and human samples demonstrated comparable migration distances, which were significantly lower than the migration distances of two primary cell types. As a platform technology, this wound healing assay may function as a new model to study migration of VSMCs within microfluidic environments.

Atherosclerosis and intimal hyperplasia are major causes of morbidity and mortality in the field of vascular diseases^1^,^2^,^3^,^4^. These processes develop secondary to endothelial injury and once this injury occurs, an essential element in the development of both these processes is the vascular smooth muscle cell (VSMC) migration^5^,^6^. Thus, understanding the mechanisms involved in VSMC migration and the development of strategies to inhibit VSMC migration have been a major focus of research^7^.

Both tissue-level and cell-level methods have been developed to study the migration of VSMCs^8^,^9^,^10^,^11^,^12^,^13^,^14^,^15^,^16^,^17^. Immunohistochemical studies of vessels from atherosclerotic patients and animal models of vessel injury report a snapshot of VSMCs in both the media and intima. In diseased or injured tissues, there are more VSMCs in the intima, attributable in part to migration of VSMCs from the media layer^8^,^9^,^10^. Although these studies on atherosclerotic human samples or animals with injured vessels can shed direct evidences on the migration of VSMCs in response to vascular injuries, the intrinsic complexities at the tissue level limit further exploration of mechanisms regulating VSMC migration.

At the cell level, there are mainly two approaches to study VSMC migrations, which are the Boyden chamber assay and the wound healing assay^7^. In the Boyden chamber assay, cultured cells are plated on the top surface of a porous membrane and migrate to the bottom surface in response to chemical gradients, which are then stained and counted^18^,^19^. In the field of VSMC migration, various chemokines including platelet derived growth factors, transforming growth factors and epidermal growth factors were located^11^,^12^,^13^. Although powerful, this approach cannot mimic the response of vascular injuries and cannot monitor the cellular migration processes under microscopy.

Meanwhile, wound-healing assays have been used to study cell migration where cells are grown to confluence and a thin “wound” is introduced by scratching with a pipette tip. Cells at the wound edge polarize and migrate into the wound space^20^,^21^. As to the studies of VSMCs, this approach has been used to track cell migration, cell-substrate adhesion forces and the healing process^14^,^15^,^16^,^17^. However, conventional wound healing assays are conducted in micro-plates and cannot reproduce local vascular injuries and reconstruct local microenvironments of VSMCs.

Microfluidics is the science and technology of manipulating and detecting fluids in the micro scale^22^,^23^. Due to dimensional comparisons with biological cells, microfluidics has been used to construct more *in vivo* like cell culture models^24^,^25^,^26^, enabling tumour^27^,^28^, neuron^29^, and vascular^30^,^31^ studies.

More specifically, microfluidics based wound healing assays have been proposed as effective tools for cell migration studies, which can mimic *in vivo* like local microenvironments more closely than conventional wound healing assays^32^,^33^,^34^,^35^,^36^,^37^,^38^,^39^,^40^,^41^,^42^,^43^,^44^,^45^. The majority of microfluidics based wound healing assays leverage the multiple laminar flows in microfluidic channels to selectively remove cells enzymatically, generating the wound with a clear boundary and monitoring corresponding cell migration^33^,^37^,^38^,^41^,^42^,^45^.

This approach was firstly demonstrated by Nie *et al.* where the migration of NIH-3T3 fibroblasts was studied^33^, which was further expanded to study various cell types including rat lung epithelial cells^41^, human umbilical vein endothelial cells^37^, human breast cancer cells (MCF-7)^38^, mouse mammary epithelial cells (CLS-1)^42^ and human alveolar epithelial-like cells (A549)^45^. However, no systematic studies of on-chip VSMC migration were previously demonstrated.

To address this issue, we proposed a microfluidic wound-healing assay enabling the quantification of VSMC migration (see Fig. 1(a–c)). In this study, gravity was used to facilitate VSMC seeding and culture (Fig. 1(d)), wound generation (Fig. 1(e)) and cell migration monitoring (Fig. 1(f)). The effects of channel geometries (variations in channel height and PDMS based channel thickness), surface modifications (fibronectin vs. collagen) and chemokines (fetal bovine serum (FBS), platelet derived growth factor BB (PDGF-BB) and tumour necrosis factor alpha (TNF-α)) on VSMC migration were investigated and compared.

In addition, the proposed microfluidic platform was used to quantify the migration of five types of VSMCs including the human aortic vascular smooth muscle cell line (T/G HA-VSMC), two types of primary aortic vascular smooth cells with human (HASMC) and rat (RASMC) origin, and VSMCs isolated from two human samples. As a platform technology, this microfluidic platform may enable the study of VSMC migration in a more physiologically relevant manner.

## Materials and Methods

### Materials

The human aortic vascular smooth muscle cell line (T/G HA-VSMC, Cat. CRL-1999) was purchased from American Type Culture Collection (ATCC, Manassas, VA, USA). Primary cell types including human aortic smooth muscle cells (HASMCs, Cat. #6110) and rat aortic smooth muscle cells (RASMCs, Cat. #R6110) were purchased from ScienCell (San Diego, CA, USA). Culture medium used in this study includes Dulbecco Modified Eagle Medium (DMEM, Hyclone) and Smooth Muscle Cell Medium (SMCM, ScienCell) while the other cell-culture reagents were purchased from Life Technologies (Thermo Fisher Scientific Inc. Waltham, MA USA). Note that supplemented culture medium represents culture medium supplemented with 10% FBS and 1% penicillin and streptomycin.

Chemokines used in this study to induce migration of VSMCs were PDGF-BB (Peprotech, Cat. #AF-100-14B) and TNF-α (Peprotech, Cat. #AF-300-01A). The materials used during device fabrication were SU-8 photoresist (MicroChem Corp., Newton, MA, USA) and 184 silicone elastomer (Dow Corning Corp., Midland, MI, USA). Fibronectin (Sigma) and collagen type I (Sigma) were used for channel surface coating.

### Human Specimen Isolation and Cell Culture

Internal mammary arteries and the ascending part of aorta were obtained from patients undergoing coronary artery bypass grafting and aortic arch replacement, respectively. All samples were obtained with the agreement of the patients and approved by the Peking University People’s Hospital Medical Ethics Committee (Beijing, China). The methods involved with human specimen isolation and cell culture were carried out in accordance with the approved guidelines.

Isolation of VSMCs from human samples was described as follows. Isolated human samples were thoroughly washed by the heparinzed PBS (50 ml PBS with 1000 units of heparin sodium) to remove blood residues, followed by the removal of adipose tissues, para-aortic lymph nodes, the adventitia and the endothelium based on sample dissection. Then samples were cut into 1 mm^2^ pieces and soaked within the enzyme solution for about 4–6 hours at 37 °C with low speed agitation to facilitate enzymatic digestion. Following the digestion step, the samples were centrifuged at 1000 rpm for 10 minutes and the cell pellets were seeded in culture flasks in DMEM supplemented with 20% FBS at 37 °C in a humidified atmosphere containing 5% CO_2_. VSMCs isolated from human samples were passaged with 0.125% trypsin and cell passage 4–6 was used for experiments.

T/G HA-VSMCs (p6–p12) were cultured with the DMEM supplemented with 10% FBS and 1% penicillin and streptomycin. HASMCs (p3–p6) and RASMCs (p3–p6) were cultured with the SMCM supplemented with 2% FBS and 1% penicillin and streptomycin.

Note that for all the VSMCs, they were cultured in 10% FBS for two passages before the migration experiments. Immediately prior to an experiment, cells were trypsinized, centrifuged and resuspended in supplemented culture medium with a concentration of 5 million cells per ml.

### Device Design and Fabrication

**Figure 1:**
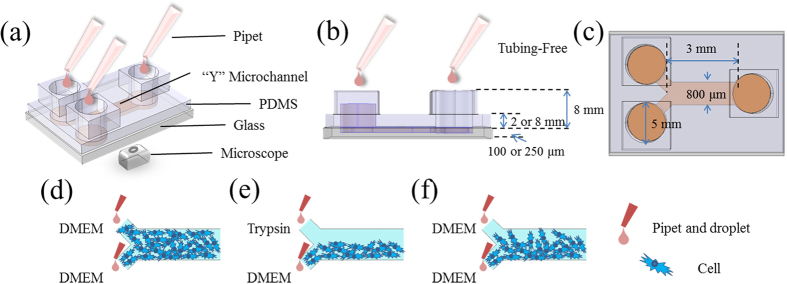
(**a**–**c**) Schematic of the tubing-free microfluidic wound-healing assay quantifying vascular smooth muscle cell (VSMC) migration (port diameter: 5 mm, channel length: 3 mm, channel width: 800 μm, channel height: 100 vs. 250 μm, PDMS layer thickness: 2 vs. 8 mm). Gravity was used to facilitate VSMC seeding and culture (**d**), wound generation (**e**) and cell migration monitoring (**f**).

Microchannels (PDMS) with channel dimensions of 3 mm in length, 0.8 mm in width were designed in this study with variations in channel height (100 μm vs. 250 μm) and PDMS thickness (2 mm or 8 mm) to investigate the effect of channel geometries on migration of VSMCs. Note that the choice of channel geometries (i.e., channel height and PDMS thickness) was based on previously reported microfluidic wound healing assays^33^,^37^,^38^,^41^,^42^,^45^. Three microchannel ports (two channel inlets and one outlet) with a diameter of 5 mm were designed in this study to facilitate liquid droplet manipulation. A total of four channels were included in one mask to characterize device operation repeatability (see Fig. 1).

**Figure 2:**
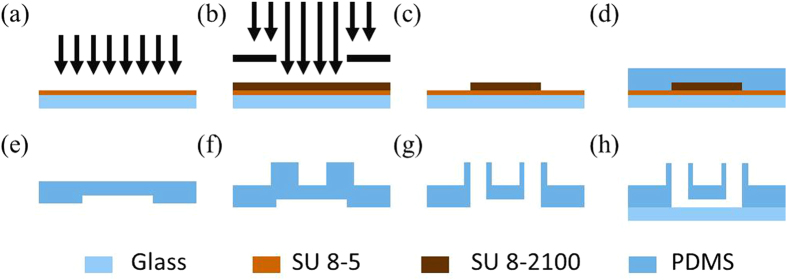
Device fabrication process including SU-8 5 seed layer fabrication. (a), mold master fabrication using SU-8 2100 (b,c), channel formation (d,e), two-layer PDMS bonding producing enhanced height around channel ports (f), through hole punching (g), and PDMS-glass bonding (h).

The PDMS device was replicated from a single-layer SU-8 mold based on conventional soft lithography (see Fig. 2). Briefly, SU-8 5 was spin coated on glass, flood exposed and hard baked to form a seed layer (see Fig. 2(a)), followed by SU-8 2100 spin coating, exposure and development (see Fig. 2(b)), forming the mold master of the microfluidic channels with a height of 100 or 250 μm (see Fig. 2(c)). To form the microfluidic device with a PDMS thickness of 2 mm, PDMS prepolymers (24 g) and curing agents (2 g) were mixed, degassed and casted on channel masters to form PDMS channels with a thickness of 2 mm (Fig. 2(d)). Furthermore, PDMS pillars with a diameter of 6 mm and a thickness of 6 mm were bonded with the patterned PDMS layers (see Fig. 2(e,f)) with through holes punched and then bonded to glass slides (see Fig. 2(g,h)).

As to the devices with a PDMS thickness of 8 mm, PDMS prepolymers (96 g) and curing agents (8 g) were mixed, degassed, poured on channel masters and baked in an oven. Cured PDMS channels with a thickness of 8 mm were then peeled from the SU-8 masters with through holes punched and bonded with glass slides.

### Device Operation and Data Analysis

In order to evaluate the device capability of generating laminar fluid flow, the microfluidic devices were first filled with DI water. Then, the solution at the outlet was removed thoroughly and the solutions in two inlets were replaced with a fluorescent solution (fluorescein isothiocyanate dextran with an averaged molecular weight of 10000 Da at 0.1 mg/mL, Sigma) and DI water at a volume of 120 μL, respectively. Time-sequence microscopic pictures of fluorescence based laminar flow within microfluidic channels were taken to evaluate the laminar flow status.

The surface coating procedure was summarized as follows: the fabricated microfluidic devices were sterilized in a hood under ultraviolet (UV) overnight, followed by surface coating of fibronectin (0.1 mg per ml) or collagen (1 mg per ml) where surface coating solutions were flushed into the microfluidic devices using micro pipets and kept within the microfluidic channels overnight (Note that the coating period (overnight) is significantly longer than what is suggested by the providers (~1 hour) and thus it is assumed that all the available spots on the channel surfaces are taken by fibronectin or collagen). Then, the coating solution was removed by aspiration and the channels were thoroughly rinsed with supplemented culture medium.

As to cell loading, supplemented culture medium solutions in three ports were removed thoroughly and replaced with cell suspension solutions of 20 μl at two inlets and 30 μl at the outlet (5 million cells per ml). After five minutes of sedimentation, each microfluidic device was placed in a petri dish containing PBS to limit evaporation and transferred to a cell incubator. Supplemented culture medium was replaced every 12 hours where solutions in three ports were removed and replaced with 60 μl fresh supplemented culture medium each.

After the formation of confluent monolayers, supplemented culture media were replaced with culture media without FBS for 24-hour starvation to synchronize the activity of vascular smooth muscle cells. Then the trypsin solution and the supplemented culture medium (120 μl) were applied at two channel inlets, respectively, to generate the wound. Using T/G HA-VSMCs as the research model, different durations (e.g., 0.5 minute, 1 minute, 1.5 minute, 2 minute and 2.5 minute) were used to generate the wound with performances compared. Following the wound generation, the trypsin solution was replaced with culture medium supplemented with different chemokines and the microfluidic devices were transferred to the incubators. Note that four groups of chemokines were used in this study, including culture medium without FBS (No FBS), culture medium supplemented with 10% FBS (FBS), 10 μg/mL PDGF-BB in supplemented culture medium (PDGF-BB) and 25 μg/ml TNF-α in supplemented culture medium (TNF-α).

Images of VSMCs with wound generation were taken on 0 hour, 3 hour, 6 hour, 12 hour and 24 hour, respectively (×10 magnification with a Olympus DP73 digital camera, Olympus Inc., Japan). Subsequently, the NIH ImageJ image analysis software was used to outline the wound areas taken by cells at 0 hour, 3 hour, 6 hour, 12 hour and 24 hour. The averaged cell migration distances at 3 hour, 6 hour, 12 hour and 24 hour were obtained by first subtracting the wound areas at 0 hour and then dividing the length of the analysed region.

Conventional wound healing assays were also conducted as a control to study the migration of VSMCs, with the procedures briefly described as follows. After the formation of confluent monolayers in 6-well plates pre-coated with fibronectin, supplemented culture media were replaced with culture media without FBS for 24-hour starvation to synchronize the activity of vascular smooth muscle cells. Then pipette tips were used to physically remove VSMCs, forming “wounds” with a width of roughly 1 mm. The steps of image collection and processing in the conventional wound healing assays were consistent with previously described procedures for the microfluidics based counterparts.

In each group, the measurement of three samples was conducted with results expressed by averages and standard deviations. ANOVA (S-N-K method, coding in Excel) was used for multiple-group comparisons where values of P <0.05 (*) and P <0.01 (**) were considered statistical significance and high statistical significance, respectively.

## Results and Discussion

The majority of microfluidics based wound healing assays request the use of external pumps to generate laminar fluid flow to selectively remove cells enzymatically. Although the use of the external pumps can provide a high-accuracy control of the laminar flow within the microfluidic devices, it is not accessed by conventional cell laboratories and the use of tubing can bring additional concerns during the step of cell loading and seeding (e.g., cell loading unevenness due to adhesion on tubing walls and unintentional flow during cell seeding resulting from tubing disturbances).

To address this issue, in this study, gravity was used as the driving force to regulate fluid flow, enabling the wound generation without the requirement of external pumps and tubing. To demonstrate the robustness of the developed approach, commercially available cell types including a transfected VSMC cell line, two primary VSMCs with human and rat origin and VSMCs isolated from two human samples were seeded into the microfluidic platform with migration distances investigated.

### Parameter Optimization in Wound Generation

The generation and maintenance of a laminar flow with a clear boundary between the trypsin solution and the culture medium is critical in the process of microfluidics based wound generation. In this study, this process is regulated by the volumes of droplets applied at the two inlets with the solution in the outlet thoroughly removed where the droplet height difference between the inlets and the outlet functions as the driving force for liquid flow. An increase in the volume of the channel inlets can lead to longer time duration to maintain the laminar flow boundary and a higher fluid stress, which is preferred in removing cells and generating the wound edge. However, if the volume of the droplet is higher than the maximal capacity of the channel inlet, it can generate different surface tensions between the channel inlets and the channel outlet, which is definitely undesirable since gravity is no longer the only power source of fluid flow. Thus, in this study, the droplet volume of 120 μl, as the upper capacity of the channel inlets (a diameter of 5 mm and a thickness of 8 mm) was used to generate the laminar flow.

**Figure 3:**
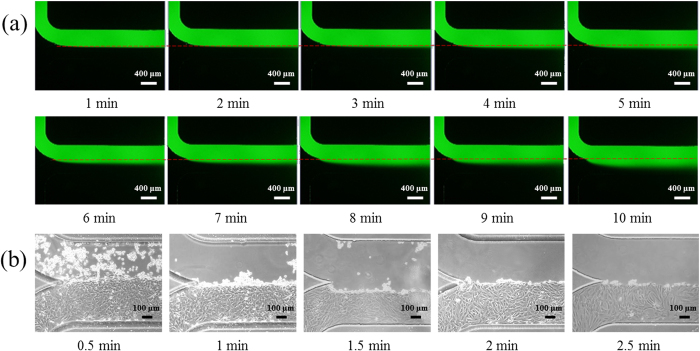
(**a**) Time-sequence microscopic pictures of fluorescence based laminar flow within microfluidic channels to mimic wound generation, indicating a stable laminar flow with a clear boundary within 5 minutes. (**b**) Time-sequence microscopic pictures of T/G HA-VSMCs under trypsin based laminar flow, suggesting that the two-minute period of trypsin flow can selectively remove cells and generate the wound.

Figure 3(a) shows time-sequence microscopic pictures of fluorescence based laminar flow within microfluidic channels to mimic the wound generation situation. It was observed that within the first five minutes, there is a clear boundary between the fluorescent solution and DI water. As time goes by, there is a decrease in the flow rate, leading to a higher diffusion of fluorescein isothiocyanate dextran molecules with a blurred boundary condition.

Whether the trypsin solution generated by gravity was strong enough to remove cells and form the wound is a concern, which needs further optimization. Using the T/G HA-VSMCs as the research model (medium: culture medium + FBS, channel height: 100 μm, PDMS thickness: 2 mm, coating: fibronectin), the effect of time duration on the removal of VSMCs was investigated.

As shown in Fig. 3(b), if the trypsin solution was neutralized within one minute (0.5 minute and 1 minute), cells were not thoroughly removed by the trypsin flow. Starting from the time duration of 1.5 minute (1.5 minute, 2 minute and 2.5 minute), cells exposed to the trypsin fluid flow were removed from the substrate clearly. Thus, in this study, 2-minute time duration was used to remove T/G HA-VSMCs. Note that in this study, the shear stress due to fluid flow was not considered as a potential factor of regulating cellular functions. This is because that the fluid flow only lasts several minutes, which is significantly lower than the time duration requested in the shear stress studies (e.g., several hours).

### Effect of Channel Geometries and Surface Coating on Migration of VSMCs

**Figure 4:**
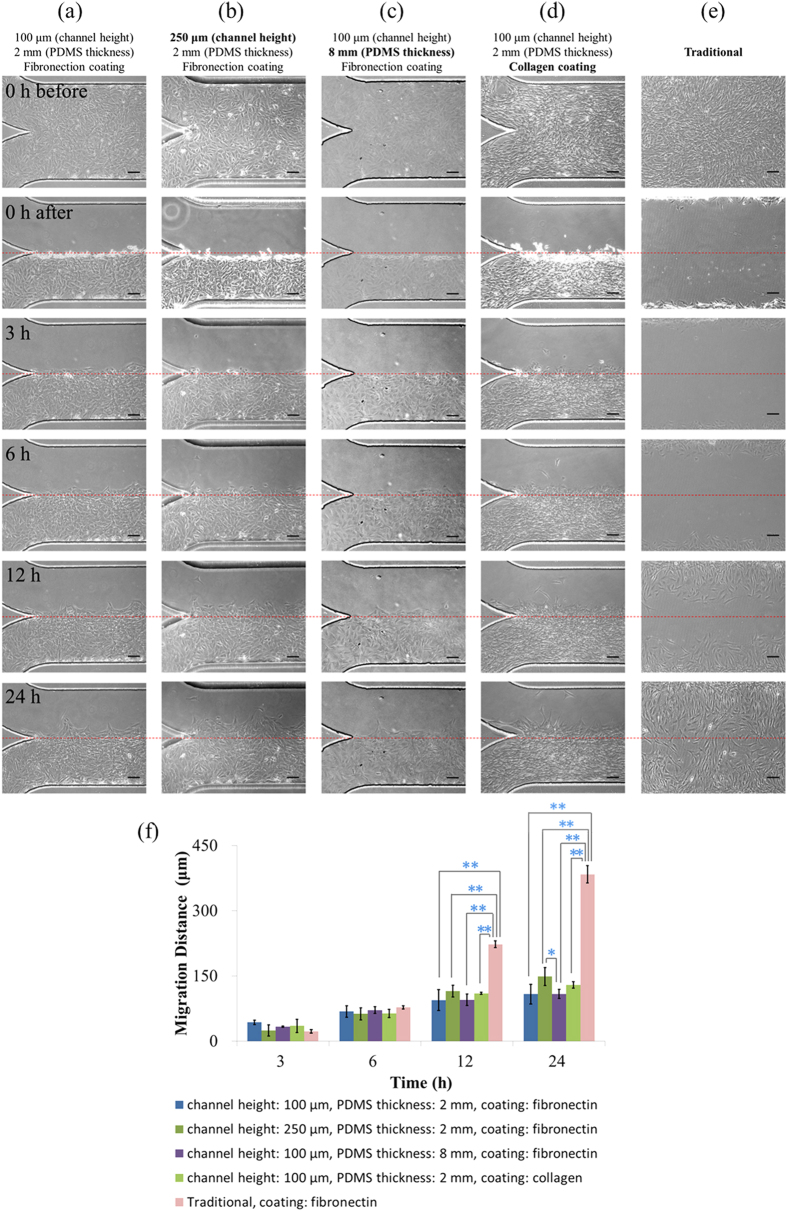
(**a**–**e**) Microscopic pictures of VSMCs in the wound-healing assay including the formation of the confluent monolayer, 0 h, 3 h, 6 h, 12 h and 24 h after wound generation with quantified migration distances shown in (**f**), in order to investigate the effects of channel geometries and surface coating on migration of VSMCs. Five groups (cell type: T/G HA-VSMC, medium: DMEM supplemented with FBS) were compared as follows: (**a**) channel height: 100 μm, PDMS thickness: 2 mm, coating: fibronectin; (**b**) channel height: 250 μm, PDMS thickness: 2 mm, coating: fibronectin; (**c**) channel height: 100 μm, PDMS thickness: 8 mm, coating: fibronectin; (**d**) channel height: 100 μm, PDMS thickness: 2 mm, coating: collagen; (**e**) traditional approach using fibronectin coating. The scale bar is 100 μm. In each group, the measurement of three samples was conducted. *represents p < 0.05 and **represents p < 0.01.

Compared to conventional wound-healing assays, microfluidic devices can provide a more *in vivo* like environment where VSMCs were exposed to limited culture medium and oxygen supplies. In order to investigate the effects of channel geometries on the migration of VSMCs, four groups of experiments were conducted (cell type: T/G HA-VSMC, medium: DMEM supplemented with FBS, coating: fibronectin, see Fig. 4). Experimental results indicate significantly lower cell migration distances within the microfluidic devices compared to traditional wound healing assays (p < 0.01), which were quantified as follows (24 hours after wound generation) 108.1 ± 22.9 μm (channel height: 100 μm, PDMS thickness: 2 mm, Fig. 4(a)), 148.9 ± 20.5 μm (channel height: 250 μm, PDMS thickness: 2 mm, Fig. 4(b)), 108.7 ± 10.5 μm (channel height: 100 μm, PDMS thickness: 8 mm, Fig. 4(c)), and 383.7 ± 19.9 μm (conventional setup, Fig. 4(e)) (see Fig. 4(f)).

More specifically, experimental results (Fig. 4(a,b,f)) show the effect of channel height on the migration of VSMCs. Within the first three hours of wound formation, the migration distances of T/G HA-VSMCs in channels with a height of 100 μm were higher than cells in channels with a height of 250 μm (43.3 ± 5.2 μm vs. 24.8 ± 12.9 μm). However, as the time goes by, T/G HA-VSMCs in channels with a higher channel height demonstrated higher migration capabilities, leading to the migration distances of 148.8 ± 20.5 μm (channel height of 250 μm) vs. 108.1 ± 22.9 μm (channel height of 100 μm) after 24 hours of wound formation. These results were consistent with previous studies of cell culture within microfluidic devices, where increases in channel height can bring a better environment for cell growth^46^.

As to the effect of PDMS thickness on cell migration, the migration distances of T/G HA-VSMCs were quantified as 108.1 ± 22.9 μm vs. 108.7 ± 10.5 μm for the PDMS thickness of 2 mm and 8 mm, respectively (see Fig. 4(a,c,f), 24 hours following wound formation). The thickness of PDMS was assumed to regulate the diffusion of oxygen, which can further affect cellular properties including cell growth and migration^47^. However, in this study, no significant difference in the cell migration distance was observed when the thickness of PDMS was increased from 2 mm to 8 mm, which may result from the following two reasons. Firstly, the thickness of PDMS is still within one centimetre and it is possible that this thickness is not high enough to function as the bottleneck of oxygen diffusion. Secondly, due to the limited channel length (3 mm in total) and large areas of channel inlets and outlets (5 mm in diameter), oxygen can effectively diffuse from the channel ports to reach the cell layer, bypassing the route of diffusion through the PDMS layer.

Extracellular matrix proteins have long been regarded as important parameters in modulating the growth and migration of VSMCs^48^. In a previous study, we investigated the effects of extracellular matrix on cell seeding and proliferation in microfluidic devices^49^. In this study, the effect of fibronectin and collagen on cellular migration was quantified and compared as follows (cell type: T/G HA-VSMC, medium: culture medium + FBS, channel height: 100 μm, PDMS thickness: 2 mm): 43.3 ± 5.2 μm vs. 35.1 ± 15.1 μm (3 Hour), 68.2 ± 12.9 μm vs. 63.8 ± 9.3 μm (6 Hour), 94.4 ± 24.0 μm vs. 110.0 ± 2.6 μm (12 Hour), 108.1 ± 22.9 μm vs. 129.4 ± 7.4 μm (24 Hour) (see Fig. 3(a,d,f)). This comparison suggests that compared to fibronectin, collagen can, to an extent, provide a better environment to promote the migration of VSMCs.

### Effect of Chemical Stimuli on Migration of VSMCs

**Figure 5:**
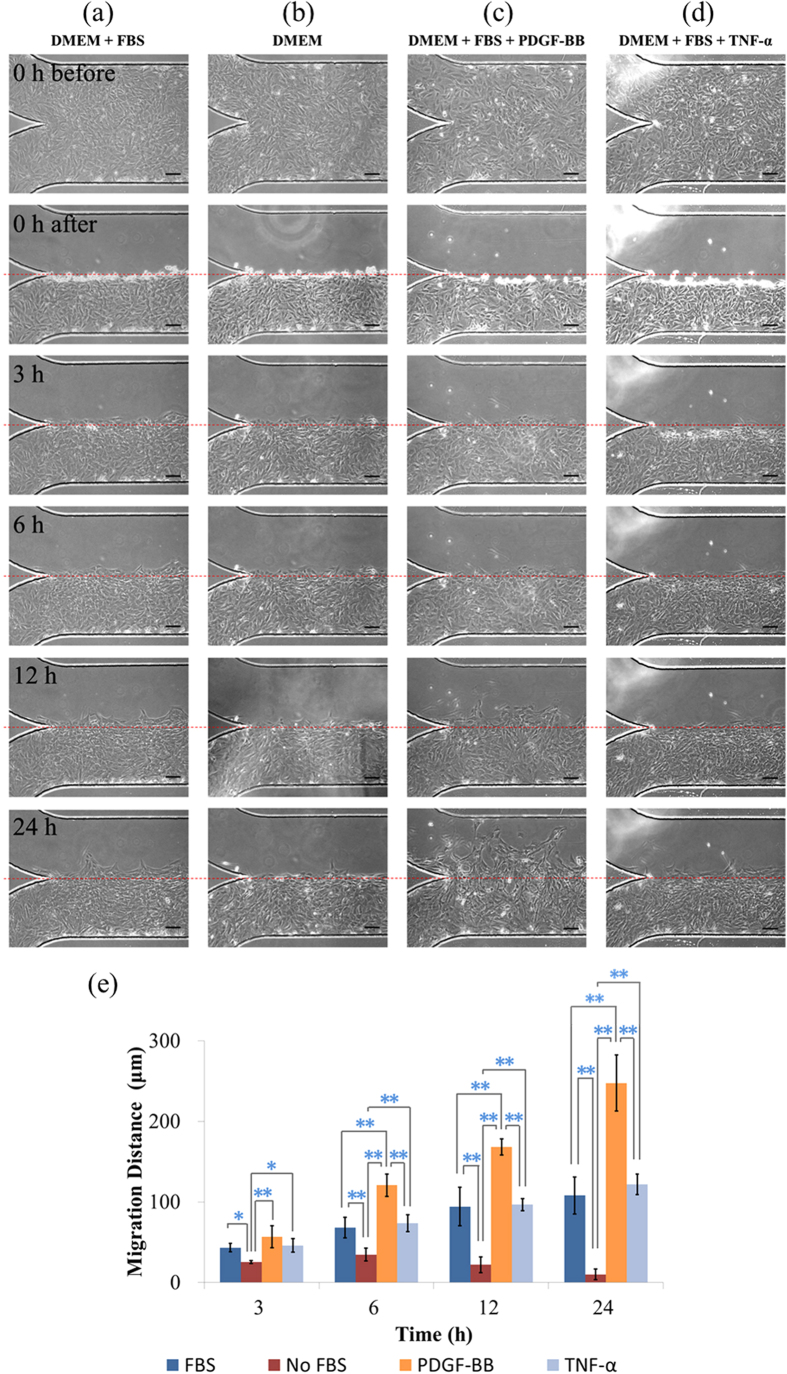
(**a**–**d**) Microscopic pictures of VSMCs in the wound-healing assay including the formation of the confluent monolayer, 0 h, 3 h, 6 h, 12 h and 24 h after wound generation with quantified migration distances shown in (**e**), in order to investigate the effect of chemokines on the migration of VSMCs. Four groups with different chemokines were compared as follows (cell type: T/G HA-VSMC, channel height: 100 μm, PDMS thickness: 2 mm, coating: fibronectin): (**a**) DMEM + FBS; (**b**) DMEM only; (**c**) PDGF-BB plus; (**d**) TNF-α plus. The scale bar is 100 μm. In each group, the measurement of three samples was conducted. *represents p < 0.05 and **represents p < 0.01.

Based on the conventional wound healing or transwell assays, various chemokines have been located to induce the migration of VSMCs^7^. In the microfluidic environments, the effects of three potential chemokines (FBS, PDGF-BB and TNF-α) on migration of VSMCs were studied (see Fig. 5, cell type: T/G HA-VSMC, channel height: 100 μm, PDMS thickness: 2 mm, surface coating: fibronectin).

Figure 5(a,b) compared the migration distances of VSMCs with and without the effect of FBS, which were quantified as 108.1 ± 22.9 μm (FBS plus) vs. 10.1 ± 6.7 μm (FBS minus) at 24 hours following the wound generation (p < 0.01) (see Fig. 5(e)). These results confirmed the positive effect of FBS on the migration of VSMCs, which is well recognized in the field of cell migration^50^.

PDGF-BB is the most potent chemoattractant for the migration of VSMCs, which has been assumed to play a major role in atherosclerosis and restenosis^11^. In the microfluidic would healing assay, a significant increase in migration distances of VSMCs due to PDGF-BB (10 μg/mL^51^) was recorded as 247.7 ± 34.8 μm (PDGF-BB plus) vs. 108.1 ± 22.9 μm (FBS) (Fig. 5(a,c,e), p < 0.01).

Besides PDGF-BB, a second chemokine TNF-α was also used in this study since TNF-α is present in atherosclerotic plaques rather than in normal vessels^52^. In previous studies, we confirmed the positive role of TNF-α in regulating the migration of VSMCs^51^. Figure 5(a,d) summarizes the experimental results with quantified migration distances of 122.0 ± 12.8 μm (TNF-α plus, 25 μg/ml^51^) vs. 108.1 ± 22.9 μm (FBS) (see Fig. 5(e)), confirming the positive role of TNF-α in inducing the migration of VSMCs.

Note that the migration distances of VSMCs induced by TNF-α are significantly lower than the migration distances induced by PDGF-BB (122.0 ± 12.8 μm vs. 247.7 ± 34.8 μm) (p < 0.01). This is because TNF-α is primarily recognized as the chemokine inducing the migration of inflammatory cells and its role in inducing VSMC migration is secondary to PDGF-BB.

### Migration Capabilities of Different VSMCs

In the previous studies of VSMC migration, VSMCs were usually isolated from self-feed mice or a single type of commercially available cells. Based on the microfluidic wound healing assay, we quantified and compared the migration of five types of VSMCs including the human vascular smooth muscle cell line (T/G HA-VSMC), two types of primary VSMCs with human (HASMC) and rat (RASMC) origin, and VSMCs isolated from two human samples (normal internal thoracic arteries vs. ascending part of aorta with aortic dissection), respectively (medium: culture medium + FBS, channel height: 100 μm, PDMS thickness: 2 mm, coating: fibronectin). Note that the choice of these cells covers the major sources of VSMCs which are currently available, including cell line, primary cells, and human samples.

**Figure 6:**
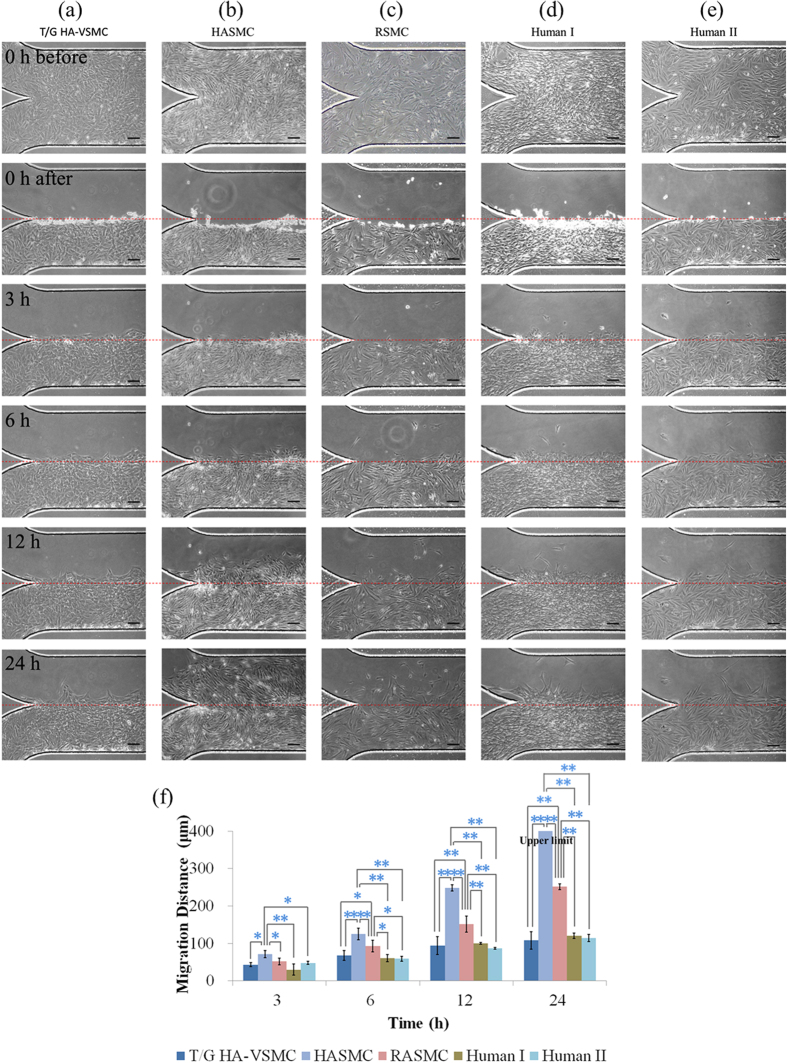
(**a**–**e**) Microscopic pictures of VSMCs in the wound-healing assay including the formation of the confluent monolayer, 0 h, 3 h, 6 h, 12 h and 24 h after wound generation with quantified migration distances shown in (**f**), as a comparison of migration capabilities of different types of VSMCs. Five types of VSMCs were compared as follows (medium: culture medium + FBS, channel height: 100 μm, PDMS thickness: 2 mm, coating: fibronectin): (**a**) T/G HA-VSMC; (**b**) HASMC; (**c**) RASMC; (**d**) VSMCs isolated from human sample I (normal internal thoracic arteries) and (**e**) VSMCs isolated from human sample II (ascending part of aorta with aortic dissection). In each group, the measurement of three samples was conducted. *represents p < 0.05 and **represents p < 0.01.

As shown in Fig. 6, significant differences (p < 0.01) in cellular migration distances among three commercially available cell types were located (24 hours following wound generation), which were quantified as 108.1 ± 22.9 μm (T/G HA-VSMC, Fig. 6(a)), >400 μm (HASMC, Fig. 6(b)), and 251.7 ± 7.5 μm (RASMC, Fig. 6(c)) (see Fig. 6(f)). Theoretically, primary cell types and cell lines should demonstrate comparable migration capabilities. However, in this study, HASMCs migrate much longer distances than T/G HA-VSMCs.

To address this issue, VSMCs isolated from the human normal internal thoracic arteries (human I) were seeded in the microfluidic platform, with quantified migration distances of 120.4 ± 7.4 μm (see Fig. 6(d,f)), demonstrating comparable capabilities with T/G HA-VSMCs. Furthermore, the migration distances of VSMCs isolated from the ascending part of aorta with aortic dissection (human II) were quantified as 114.8 ± 9.7 μm on 24 hours following the wound generation (see Fig. 6(e,f)). Note that in aortic dissection, VSMCs were characterized with phenotype transition and apoptosis without significant variations in cellular migration^53^.

In summary, T/G HA-VSMCs and VSMCs isolated from two human beings demonstrated comparable migration distances, which are significantly lower than the migration distances of primary aortic smooth muscle cells (HASMC). It was speculated that the HASMCs in this study were obtained from human arterials suffering atherosclerosis rather than normal aortas and therefore they demonstrated significantly higher capabilities in migration.

## Conclusions and Future Work

This paper proposed a tubing-free microfluidic wound healing assay enabling the quantification of VSMC migration. The effects of geometries on the migration of VSMCs were explored, revealing the positive role of channel height on cell migration while the role of PDMS thickness in regulating cell migration is negligible. Compared to fibronectin, the surface coating of collagen was demonstrated to induce more migration of VSMCs. Three chemokines including FBS, PDGF-BB and TNF-α were demonstrated to promote cell migration and the migrations of five types of VSMCs were quantified and compared. T/G HA-VSMCs and VSMCs isolated from two human samples indicated comparable migration capabilities while the migration distances of primary aortic smooth muscle cells (HASMC and RASMC) were significantly higher.

From the technical development, future work will focus on the scale up of the current microfluidic devices. By defining the positions of channel inlets and outlets to be compatible with 96-well or even 384-well micro plates, high throughput characterization of cellular migration in the microfluidic wound-healing assay can be realized. As to the cell types under measurement, future studies will focus on the culture of human atherosclerotic plaques and quantify the migration distances of diseased VSMCs.

## Additional Information

**How to cite this article**: Wei, Y. *et al.* A Tubing-Free Microfluidic Wound Healing Assay Enabling the Quantification of Vascular Smooth Muscle Cell Migration. *Sci. Rep.* **5**, 14049; doi: 10.1038/srep14049 (2015).
